# Reliability of a Repeated High-Intensity Effort Test for Elite Rugby Union Players

**DOI:** 10.3390/sports8050072

**Published:** 2020-05-22

**Authors:** Adrien Vachon, Nicolas Berryman, Iñigo Mujika, Jean-Baptiste Paquet, Tony Monnet, Laurent Bosquet

**Affiliations:** 1 Lab. MOVE (EA6314), Faculty of sport sciences, University of Poitiers, 86000 Poitiers, France; berryman.nicolas@uqam.ca (N.B.); adrien.vachon@univ-poitiers.fr (A.V.); 2 Stade Rochelais Rugby, Department strength and conditioning, 27 Avenue du Maréchal Juin, 17000 La Rochelle, France; jbpaquet@staderochelais.com; 3 Département des Sciences de l’activité physique, 141 Avenue du President Kennedy, Université du Québec à Montréal, Montréal, QC H2X 1Y4, Canada; 4 Institut national du sport du Québec, Pierre de Coubertin, Montréal, QC H1V 3N7 Canada; 5 Department of Sports Studies, Bishop’s University, College, Sherbrooke, QC J1M 1Z7 Canada; 6 Department of Physiology, Faculty of Medicine and Nursing, University of the Basque Country, Leioa, Basque Country; inigo.mujika@inigomujika.com; 7 Exercise Science Laboratory, School of Kinesiology, Faculty of Medicine, Universidad Finis Terrae, H97R+8J Santiago, Chile; 8 Prime Institute, CNRS-University of Poitiers-ENSMA, UPR 3346, 86360 Chasseneuil du Poitou, France; tony.monnet@univ-poitiers.fr; 9 Department of kinesiology, University of Montreal, succ. centre ville. Montreal, QC H3C 3J7, Canada

**Keywords:** sprints, tackles, repeated efforts, team sports, rugby union

## Abstract

This study aimed to adapt a repeated high-intensity effort (RHIE) test to the rugby union physical demands and assess both sprint time and tackle indices reliability. Following a familiarization session, sixteen elite rugby union players completed two RHIE tests consisting of 12 × 20 m sprint + tackle. Total sprint time and total g-force during tackling, average sprint time and average g-force as well as percentage decrement_sprint time_ and percentage decrement_tackle_ were considered for the analysis. Sprint time indices showed high to very high absolute and relative reliability (intraclass coefficient correlation (ICC) = 0.95, Standard Error Measurement (SEM) = 1.30%; ICC = 0.95, SEM = 1.44%; ICC = 0.73, SEM = 23.0%, for total sprint time, average sprint time and percentage decrement_sprint time_, respectively). Tackle indices showed moderate to high reliability (ICC = 0.54, SEM = 16.5%; ICC = 0.61, SEM = 15.6%; ICC = 0.71, SEM = 12.3%, for total g-force, average g-force and percentage decrement_tackle_, respectively). The RHIE test provides reliable measures of sprint time and tackle indices. Tackle indices should be used as a validation criterion of the test, whereas total time should be considered as the test final result.

## 1. Introduction

Rugby union is an opposition game between two teams of 15 players, generally classified as “Forwards” and “Backs”, with major differences between them in terms of physical and physiological requirements during a match. Forwards cover less distance than backs (i.e., around 5500 vs 6500 m, respectively) and a higher proportion of low-intensity distance (6–12 km·h^−1^) due to a higher involvement in efforts like tackles, rucks or mauls [1,2]. In contrast, Backs cover more distance at a high intensity (i.e., >20 km·h^−1^) [3], and perform more (i.e., 9 ± 4 vs. 5 ± 4 in Forwards) and longer (i.e., 20 vs 15 m in Forwards) sprints [4,5]. In spite of these differences, the relative distribution of distance covered at different velocities during a game is similar between Forwards and Backs, suggesting that acceleration and maximal speed qualities are important determinants of performance for both playing positions [5]. Due to the importance of these high-intensity efforts and their succession over the duration of a match, repeated sprint ability (RSA) is a major physical determinant of performance in rugby [4]. 

In addition to acceleration and sprints, rugby union also consists in a repetition of high-intensity contact [4]. Recent studies suggest that these bouts of high-intensity contact could affect a player’s running ability. Indeed, Johnston et al. [6] assessed the running performance (distance and intensity) of rugby league players during small-sided games with variable contact bouts (i.e., 1, 2 or 3 × 5 s wrestle periods) and observed greater reduction in running intensity with multiple contacts. Furthermore, it seems that when the contact demands become higher during small-sided games, players exhibiting better running performance are not those with the best high-intensity-running ability [6]. The addition of multiple contacts may cause an additional fatigue which may amplify the muscular limiting factors and increase the implication of central fatigue reported with RSA [7] leading to a pacing strategy adopted by players [6].

Therefore, some authors consider that the ability to repeat high-intensity efforts (RHIE) is more discriminant than the RSA [8]. The RHIE ability, within the context of rugby union, is defined as three or more intense accelerations (>2.79 m·s^−2^), sprints (>5 m·s^−1^), or contact efforts (i.e., tackle, ruck) with less than 21 s of recovery between efforts [9,10,11]. During a rugby union match, Forwards are generally more exposed to RHIE bouts than Backs (i.e., 11–18 vs. 2–18 RHIE). Independent of the playing position, RHIE bouts are generally composed of sprints and physical tasks. Indeed, Forwards are involved in longer RHIE bouts, mostly composed of scrums, rucks or mauls (≈50%) and sprints (≈30%); Backs’ RHIE bouts are mostly composed of sprints (≈45%) and tackles (≈35%) [11]. Studies suggests that an RHIE bout can be considered as soon as two efforts (i.e., instead of three) are performed, due to their higher occurrence during a game [12]. Furthermore, assessing the reliability of this kind of test is important since it allows staff to have a better knowledge of the metrological properties of the test, thereafter to determine the minimum difference to be considered real, which is the key value when assessing player improvements.

To the best of the authors’ knowledge, only three studies [8,13,14] have tried to evaluate sport-specific RHIE ability with a test design based on time-motion analysis of the game. The experimental approach consists of introducing a tackle task in an RSA test and then assessing sprint time indices as the result of the test. However, existing tests seem to have some limitations. The test proposed by Austin et al. [13] consists of a repetition of sprints (i.e., 3 × 20 m) in a first area, followed by a repetition of “sled shuttle” in a second area, which seems difficult to implement with a high number of players. Johnston et al. [8] developed a test which apparently corresponds to the training constraints, but did not assess the intensity of the tackle task during the test. It appears necessary to evaluate both sprint time and tackle intensity to better represent the demands of the game. This was the strategy adopted by Gabbett et al. [14] but the test was designed to fit the demands of rugby league matches, which are different from those in rugby union competitions [11]. 

In order to assess the RHIE ability in rugby union players, a test was updated from its original version [8] by including a longer active recovery between efforts and by increasing the starting time from 20 to 30 s to better represent work-to-rest ratios experienced during rugby union match play [3,11,15,16]. It also met the criteria of an RSA test as described by Girard et al. [7].

The aim of this study was to verify reliability properties of this sport-specific RHIE test including both sprint time and tackle indices. 

## 2. Methods

### 2.1. Participants

Fourteen elite rugby union players from the same Top 14 (French professional club competition) club participated in this study. Two participants withdrew from the study due to injury. The final sample size was 14 players (age: 19.9 ± 1.0 y; height: 183.3 ± 6.5 cm; body mass: 92.5 ± 12.6 kg). Participants were members of the U21 team, playing at the top national level, but regularly joining the professional team. All players received a clear explanation of the study, as well as a presentation of the risks and benefits associated to their participation. Written consent was obtained from the players prior to their inclusion in the study.

### 2.2. Experimental Design

Following a thorough briefing including a presentation of the protocol and a question period, all players gave their written informed consent to participate. Participants completed two RHIE tests separated by 7 days, at the same time of the day. A familiarization session was organized one week before the beginning of the study to decrease the magnitude of a possible learning effect. During the familiarization session, participants completed both a warm-up and the RHIE test in the exact same conditions as during experimental sessions. Coaches were asked to decrease the volume of strenuous training the day before each test. Participants were asked to maintain their normal diet and to arrive fully hydrated to the testing sessions, at least three hours after their last meal.

### 2.3. Exercise Testing

#### 2.3.1. Warm Up

Participants performed the same standardized dynamic warm-up at the beginning of each testing session. Briefly, this 10-min warm-up consisted of progressive athletic drills, progressive upper body activation, 3 submaximal shuttle sprints with tackle and 1 maximal shuttle sprint with a tackle. After completion of the warm-up, participants had a 4-min passive recovery period before the RHIE test.

#### 2.3.2. Repeated High Intensity Efforts (RHIE) Test

**Figure 1:**
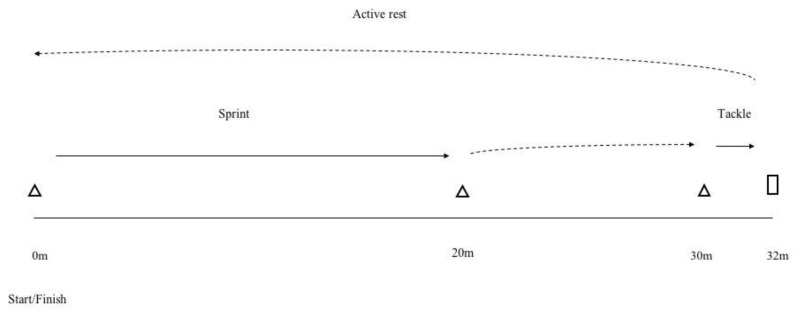
Schematic representation of the RHIE test.

The RHIE test (Figure 1) was derived from Johnston et al. [8] and consisted of 12 repetitions of a 20-m sprint immediately followed by a tackle with repetitions starting every 30 s. The clock started upon the initiation of the first sprint, then the participant performed the sprint and waited at the 30 m mark until the clock reached 10 s, at which point they made the tackle and then used the remaining time (≈15 s) to jog back to the start line and get ready for the next sprint.

The test was performed on an indoor synthetic rugby pitch. Participants were instructed to perform each sprint and each tackle at maximal intensity. Each 20-m sprint was initiated from a three-point stance “ready” standing position, 0.3 m behind the starting line. Performance of the last warm-up sprint was assessed and used as an individual sprint reference. To avoid pacing, participants had to run the first two sprints of the test at or faster than 95% of the individual sprint reference [17]. All the participants fulfilled this criterion. To perform the tackle, participants were instructed to be in an immobile position, one hand on the ground at a landmark placed at 30 m and initiate the tackle 10 s after the starting clock. The tackle involved accelerating forward 2 m and then hitting a tackle bag (Rhino tackle bag senior, 13 kg; 75cm (h) × 45cm (d); Rhino Global Limited, UK) with the highest intensity possible. Participants were asked to grip the tackle bag with both arms until the tackle was completed. Participants received clear feedback about elapsed time every 10 s while jogging back to the starting line to ensure they would be ready for the next repetition within the allocated time.

### 2.4. Data Analysis

#### 2.4.1. Sprint Performance

Time was measured to the nearest tenth of a second using photocell gates (Witty Wireless Training Timer, Microgate Corporation, Italy) placed beside (gate with 3-m width) the start and the finish lines, 1 m above the ground. No reliability statistics were found about this device.

Total sprint time, (i.e., the sum of the 12 sprint times), average sprint time, and the ratio between total sprint time and the number of repetitions were computed. Fatigue was assessed by the percentage decrement score [18], calculated as follows (Equation (1)):

(1) $${\mathrm{Percentage}\;\mathrm{decrement}}_{\mathrm{sprint}}=\left(100\times \frac{\mathrm{Total}\;\mathrm{sprint}\;\mathrm{time}}{\left(12\times \mathrm{Fastest}\;\mathrm{sprint}\;\mathrm{time}\right)}\right)-100$$

#### 2.4.2. Tackle Performance

Acceleration during the tackle was measured using a three-axis accelerometer with a sampling frequency of 100 Hz and a full range of 160 m·s^−2^ (MTw Awinda, Xsens Technologies, Enschede Netherland), placed in a small pocket located in each player’s shirt, on the upper back between the two scapulae. Raw data were downloaded using the software provided by the manufacturer (MT software Suite 4.6, Xsens Technologies, Enschede, Netherland) and subsequently corrected for gravity. No reliability statistics were found about this device. A zero-lag, low-pass Butterworth digital filter set at 12 Hz was applied in a customized MatLab program (Matlab R2018b, MathWorks, Natick, MA, USA) to remove high-frequency noise from the raw data [18]. The total magnitude of impact at time *t* was calculated by combining the 3 plane accelerations, as follows (Equation (2)):

(2) $${\mathrm{Acceleration}}_{\mathrm{Total}}=\sqrt{(a_{x}^{2}+a_{y}^{2}+a_{z}^{2}){}_{t}}$$

where a_x_ = forward acceleration, a_y_ = sideways acceleration, a_z_ = upwards acceleration, t = time. 

Acceleration was then converted in *g*-force (1 g = 9.8 m·s^−2^) [19]. Total amount of g-force, (i.e., the sum of the g-force measured during each of the 12 tackles), average g-force, and the ratio between total amount of g-force and the number of repetitions were computed. Fatigue was assessed by the percentage decrement score (Equation (1)).

#### 2.4.3. Combined Performance

To assess the participants’ overall capacity to repeat high intensity efforts involving sprints and tackles, we computed a combined indicator accounting for the performance in both components of the test, calculated as follows (Equation (3)):

(3) $${\mathrm{Percentage}\;\mathrm{decrement}}_{\mathrm{combined}}=\frac{{\mathrm{Percentage}\;\mathrm{decrement}}_{\mathrm{sprint}}+{\mathrm{Percentage}\;\mathrm{decrement}}_{\mathrm{tackle}}}{2}\;$$

### 2.5. Statistical Analysis

Standard statistical methods were used for the calculation of means and standard deviations. Normal Gaussian distribution of the data was verified by the Shapiro–Wilk test and homoscedascticity by a modified Levene Test. Systematic bias, which refers to a general trend for measurements to be different in a particular direction between repeated tests [20], was assessed with a student’s t test for dependent samples, or with a Wilcoxon test when at least one of the two underlying hypotheses were not satisfied (i.e., normality of the distribution and homoscedasticity). The magnitude of the difference was assessed by the Hedges’ *g* (g), which was considered small (0.2 < |*g*| < 0.5), moderate (0.5 < |*g*|< 0.8), or large (|*g*| > 0.8) [21]. Relative and absolute reliability, which represents the degree to which individuals maintain their position in a sample with repeated measures and the degree to which repeated measurements vary for individuals [20], were assessed with the intraclass correlation coefficient (ICC; model 2, 1) and the standard error of measurement (SEM), respectively. The SEM was calculated as follows:

(4) $$\mathrm{SEM}=\sqrt{{\mathrm{MS}}_{E}}$$

where MS_E_ is the mean-squared error.

Both the ICC and the SEM were computed from the breakdown of a two-way ANOVA (trials × subjects) with repeated measures. We considered an ICC over 0.90 very high, between 0.70 and 0.89 high and between 0.50 and 0.69 moderate [22]. Standard error measurement can also be used to determine the minimum difference to be considered real (MD), which represents the limit under which the observed difference is within what we might expect to see in repeated testing just attributed to the noise in the measurement [23]. The MD was calculated as follows:

(5) $$\mathrm{MD}=\mathrm{SEM}\times 1.96\times \sqrt{2}$$

where SEM is the standard error of measurement computed from Equation (4).

Statistical significance was set at *p* < 0.05 for all analyses. All calculations were made with Statistica 6.0 (Statsofts, Tulsa, OK, USA).

## 3. Results

**Figure 2:**
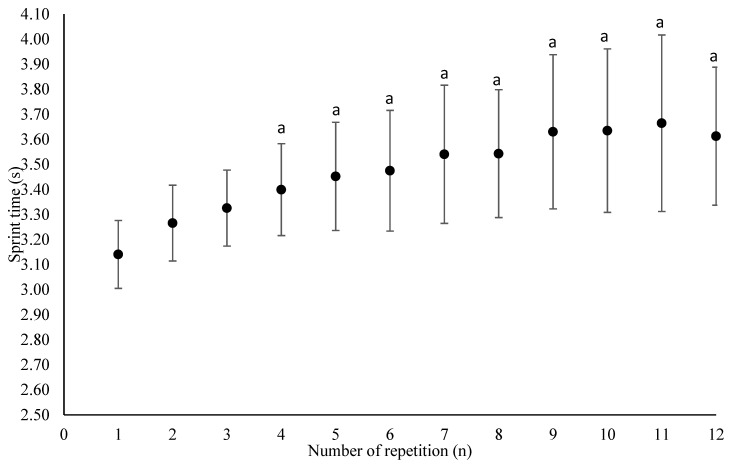
Sprint time kinetic during the repeat high-intensity efforts (RHIE) test. ^a^ Significantly different from Test 1.

**Figure 3:**
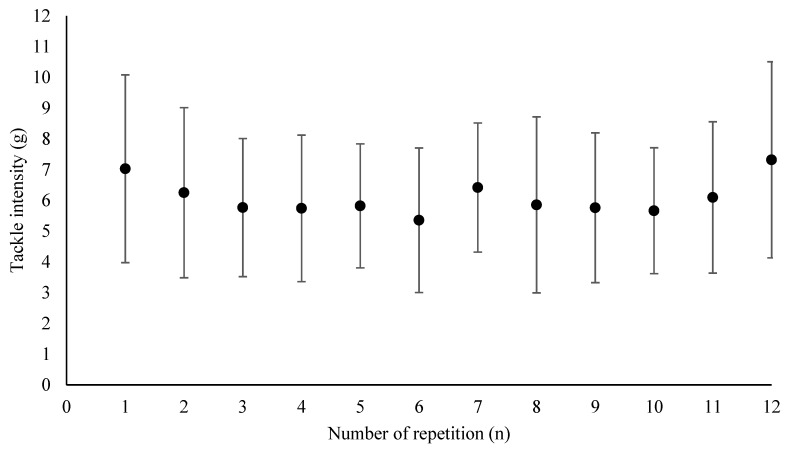
Tackle g-force kinetic during the RHIE test.

Kinetics of sprint time and tackle performance are presented (mean and standard deviation of Test 1 and 2) in Figure 2 and Figure 3, respectively. Sprint performance decreased over time with a statistical difference from the 1st repetition occurring after the 4th repetition (*p* < 0.05). Tackle performance remained stable during the test. Reliability results are presented in Table 1. The percent decrement_sprint time_ was similar between Tests 1 and 2 and showed high relative (ICC = 0.73) and poor absolute reliability (SEM = 23.0%; MD = 63.6%). A trivial but systematic bias (or learning effect) was found for total sprint time and average sprint time (*p* < 0.05; *g* = −0.17). Both indices displayed very high relative (ICC = 0.95 and 0.95, respectively) and very good absolute reliability (SEM = 1.30% and 1.44%, respectively; MD = 3.59% and 3.99%, respectively).

Concerning tackle performance, a small and systematic bias was found between test and retest values for percent decrement_tackle_ (*p* < 0.05; *g* = 0.46), together with a high-to-moderate relative and absolute reliability (ICC = 0.71, SEM = 12.3%; MD = 34.2%). Regarding the total amount of g-force and average g-force, we found no systematic bias between test and retest values, as well as a moderate relative (ICC = 0.54 and 0.61, respectively) and a moderate absolute reliability (SEM = 16.5% and 15.6%, respectively; MD = 45.9% and 43.4%, respectively).

Regarding the combined index, we found no systematic bias, together with a high relative and a good absolute reliability (ICC = 0.71, SEM = 11.9%; MD = 32.9%).

## 4. Discussion

The aim of this study was to assess the reliability of new sport-specific RHIE indices using both sprint and tackle performances. The main results were 1) sprint time indices showed high-to-very high relative and absolute reliability in our sample of elite players, and 2) in addition to a poor-to-moderate reliability, the kinetics of tackle performance questioned its usefulness in the assessment of RHIE. 

### 4.1. Test Reliability

As can be seen in Table 1, a systematic bias could be observed between Tests 1 and 2 on the total sprint time and average sprint time indices of the RHIE test. Despite the implementation of a familiarization session, this bias showed that a learning effect still occurred after three trials of the same protocol. This contrasts with previous studies indicating that no familiarization session was needed to assess repeated sprinting performance [24]. As reported by Ploutz-Snyder and Giamis [25], experienced athletes seem to require more familiarization sessions to achieve the same absolute consistency of measurement. For that reason, we suggest at least two familiarization sessions to assess RHIE with elite rugby union players.

A classical measure of relative reliability is the ICC (2, 1). The higher the ICC, the better the relative reliability, and the lower the influence of the measurement error. We observed a high-to-very high relative reliability for all sprint time indices. Values were in accordance with those previously reported in a comparable RHIE test in rugby league with high (ICC = 0.82) and very high (ICC = 0.91) relative reliability for total time and percentage decrement, respectively [8]. 

To our knowledge, no study has investigated the relative reliability of tackle indices during a repeated effort test. We observed a moderate-to-high reliability and confirmed the better reliability of total and average indices (i.e., total and average g-force) previously observed for sprint performance.

A classical measure of absolute reliability is the SEM, which provides an index of the expected trial-to-trial noise in the data [26], often complemented with the MD.

The SEMs found in this study for total and average sprint time (<1.44%) were similar to values reported for RSA tests (SEM < 1.8%) [27] whereas decrement_sprint time_ SEM of 23.0% was similar to 22.3% coefficient variation (CV) reported with soccer players [27]. Furthermore, in accordance with previous studies assessing both RSA [27] and RHIE [8], percentage decrement was found to be the least reliable parameter.

Despite efforts made to increase standardization (i.e., imposed static phase before the tackle, one hand in contact with the ground, and use of a small tackle bag to limit the variability of the impact zone on the bag), SEM of tackle indices were higher than those of speed indices (i.e., >15.6%) for total and average g-force. This is probably due to a combination of factors including the nature of the tackle movement and the equipment used. Indeed, the tackle is a complex movement where tackling technique can highly influence tackle efficiency [28]. Furthermore, tackle movement was suggested to have higher-frequency content characteristics than other contact-sport movements usually assessed [29], leading to an increase in the accelerometer’s signal-to-noise ratio [18]. 

In an attempt to assess tackle intensity during a similar RHIE test, Gabbett et al. [14] used the PlayerLoad^TM^, which is automatically calculated by the manufacturer and reported as 0.5% CV’s. Due to the high difference in terms of equipment and method reported, any comparison between both results seemed difficult.

A new combined index was also developed in this study with the aim of highlighting the players’ capacity to repeat both sprints and tackles at a high intensity. The percentage decrement_combined_ represents the overall capacity of the player to maintain both speed and tackle intensity despite the accumulation of fatigue over the repetitions. Considering that this new index is the average of percentage decrement_sprint time_ and percentage decrement_tackle_, we could expect relative reliability of percentage decrement_combined_ to be the average relative reliability of its two constituting measures. The same rationale applies to SEM and MD. However, despite the high relative reliability reported, it seems difficult to use this new combined index properly, due to the kinetics of the sprint time and tackle acceleration. Indeed, while sprint time decreased over time, tackle acceleration was maintained, and displayed a high variability. In this context, it seems difficult to apply the rationale of the percent decrement score.

### 4.2. Test Interpretation 

Despite efforts made in the tackle phase standardization, the high variability of the tackle technique, an unfavorable accelerometer signal-to-noise ratio, and possible interfering movement of the accelerometer in the shirt all led to a high variability in the acquisition of the acceleration data. In addition, contrary to what was expected (i.e., a decrease in performance with time), g-force kinetics were linear, suggesting that tackle intensity was maintained over time with a high standard deviation (Figure 3). 

Because of these limitations, we could not interpret tackle performance in the same way as sprint performance (i.e., using total g-force and percentage decrement_tackle_). However, we consider this information should be accounted for in the overall RHIE performance. That is why we suggest that, if triaxial accelerometer technology is available, the tackle performance should be used as a validation criterion. As previously underscored, it seems difficult to assert that a player gives his best on every tackle since the high variability make utilization of raw data difficult. However, to ensure that a player produces a steady effort on the tackle task, which influences the following sprint, the athlete’s g-force CV should be less than the MD of the same index [23]. Then, sprint time indices could be interpreted as the results of the RHIE test. Based on our results, athletes’ CV of the total g-force index has to be less than 45.9% and 34.2% for percentage decrement_tackle_ (Table 1). If these observations are verified, the test can be considered valid, and sprint time indices can be interpreted. However, if a triaxial accelerometer is not available, we suggest considering only the sprint time indices. But then, the importance of providing a maximal effort for each tackle should be emphasized when instructions are given to athletes.

We suggest that the main information derived from this test is the total sprint time. Indeed, this index reflects well the sprint speed of a player [7], since quickest players over 20m are also those who perform best in multiple sprints [13], and this is what truly matters on the field. After some repetitions of the RHIE test, a player with a higher maximal velocity who shows a lower capacity to resist fatigue is still more performant than a more resistant but slower player. Considering absolute reliability of the different indices, we suggest that a change in performance higher than 3.6% can be considered real (Table 1).

Nonetheless, preserving a high maximal speed while improving the ability to reproduce efforts over time is also interesting for the overall performance, and in this sense percentage decrement_sprint time_, as it informs about a player’s ability to delay fatigue and recover during the active phases of the test [7], can be used as complementary information, despite lower relative and absolute reliability.

#### Practical Applications 

The RHIE test could be used by practitioners to evaluate and assess the RHIE ability of rugby union players. Tackle indices should be used as a validation criterion of the test, whereas total time should be considered as the final result of the test, with percentage decrement_speed_ as an indicator of an athlete’s ability to resist fatigue over time. The minimum to be considered real is 3.59% for total sprint time. At least two familiarization sessions should be performed to minimize learning effects.

## 5. Conclusions

The aim of this study was to verify reliability properties of a new sport-specific RHIE test including both sprint time and tackle indices. Using commercially available technology and without altering the ecological aspect of the tackle, utilization of the tackle intensity as the main outcome of the test did not seem possible. We concluded that the RHIE test provided reliable measures of sprint time indices. This test could therefore be used by practitioners to evaluate and assess the RHIE ability of rugby union players.

Additional studies are now required to verify validity parameters of this test, either by implementing interventional studies or by assessing the association with physiological factors underlying the RHIE ability.

## Figures and Tables

**Table 1:** RHIE test indices reliability.

Parameter	Test 1 (mean ± SD)	Test 2 (mean ± SD)	Hedge’s g (g)	ICC	SEM (%)	MD (%)
**Sprint Time Indices**						
**PD (%)**	9.18 ± 4.24	8.77 ± 3.53	−0.10	0.73	23.0	63.6
**TST (s)**	41.91 ± 2.62	41.45 ± 2.54 ^a^	−0.17	0.95	1.30	3.59
**AST (s)**	3.49 ± 0.22	3.45 ± 0.21	−0.17	0.95	1.44	3.99
**Tackle Indices**						
**PD (%)**	37.33 ± 8.19	42.58 ± 11.36 ^a^	0.46	0.71	12.3	34.2
**TST (g)**	77.62 ± 19.67	70.02 ± 16.70	−0.39	0.54	16.5	45.9
**AST (g)**	6.47 ± 1.64	5.88 ± 0.46	−0.35	0.61	15.6	43.3
**Combined Index**						
**PD (%)**	23.25 ± 5.19	25.67 ± 6.01	0.40	0.71	11.9	32.9

ICC = Intraclass coefficient correlation; SEM = Standard error of measurement; MD = Minimum difference to be considered real; PD = Percentage decrement; TST = Total sprint time; AST = Average sprint time; ^a^ Significative difference from Test 1 with *p* < 0.054.
